# Music and Metronomes Differentially Impact Motor Timing in People with and without Parkinson's Disease: Effects of Slow, Medium, and Fast Tempi on Entrainment and Synchronization Performances in Finger Tapping, Toe Tapping, and Stepping on the Spot Tasks

**DOI:** 10.1155/2019/6530838

**Published:** 2019-08-18

**Authors:** Dawn Rose, Yvonne Delevoye-Turrell, Laurent Ott, Lucy E. Annett, Peter J. Lovatt

**Affiliations:** ^1^Lucerne University of Applied Sciences & Arts, Zentralstrasse 18, CH-6003 Lucerne, Switzerland; ^2^University of Hertfordshire, Department of Psychology and Sport Sciences, School of Life and Medical Sciences, College Lane, Hatfield, Hertfordshire AL10 9AB, UK; ^3^Université de Lille, SCALab, UMR 9193–CNRS, Villeneuve d'Ascq, France

## Abstract

**Introduction:**

Rhythmic auditory stimulation (RAS) has successfully helped regulate gait for people with Parkinson's disease. However, the way in which different auditory cues and types of movements affect entrainment, synchronization, and pacing stability has not been directly compared in different aged people with and without Parkinson's. Therefore, this study compared music and metronomes (cue types) in finger tapping, toe tapping, and stepping on the spot tasks to explore the potential of RAS training for general use.

**Methods:**

Participants (aged 18–78 years) included people with Parkinson's (*n* = 30, Hoehn and Yahr mean = 1.78), older (*n* = 26), and younger adult controls (*n* = 36), as age may effect motor timing. Timed motor production was assessed using an extended *synchronization-continuation* task in cue type and movement conditions for slow, medium, and fast tempi (81, 116, and 140 mean beats per minute, respectively).

**Results:**

Analyses revealed main effects of cue and movement type but no between-group interactions, suggesting no differences in motor timing between people with Parkinson's and controls. Music supported entrainment better than metronomes in medium and fast tempi, and stepping on the spot enabled better entrainment and less asynchrony, as well as more stable pacing compared to tapping in medium and fast tempi. Age was not confirmed as a factor, and no differences were observed in slow tempo.

**Conclusion:**

This is the first study to directly compare how different external auditory cues and movement types affect motor timing. The music and the stepping enabled participants to maintain entrainment once the external pacing cue ceased, suggesting endogenous mechanisms continued to regulate the movements. The superior performance of stepping on the spot suggests embodied entrainment can occur during continuous movement, and this may be related to emergent timing in tempi above 600 ms. These findings can be applied therapeutically to manage and improve adaptive behaviours for people with Parkinson's.

## 1. Introduction

Studies comparing people with and without Parkinson's disease suggest it is the loss of the dopamine-producing cells in the substantia nigra in the basal ganglia that results in the impairment of time perception and internally generated timed motor production abilities [1–7]. Although medication regimens help manage symptoms, they are not necessarily effective for improving deficits in gait, such as shuffling, step irregularity, freezing, and postural instability, for example [8, 9]. Such difficulties with walking are detrimental to the quality of life experienced by people with Parkinson's, not least because the deficits often lead to falls, which can in turn contribute to further physical and psychological health problems [10, 11]. Consequently, finding adjunct therapies to improve gait is a priority for Parkinson's-related research [12–14].

One avenue of investigation, based on findings from neuroimaging studies, has focused on how external sounds can prime the movement areas in the brain for action [15–18]. Researchers in neurologic music therapy have operationalized this as rhythmic auditory stimulation (RAS [19]). The therapeutic strategy involves recruiting the connections between the auditory and motor systems by using metronomes or rhythmically enhanced familiar music (commonly a metronome embedded into the music) to provide the external cues to improve gait. These improvements are manifested in observable positive outcomes such as regulating cadence and increasing gait velocity and stride length (e.g., [20] and see [13, 17, 21, 22] for reviews). The phenomena enabling these beneficial changes include *entrainment*, *synchronization*, and *pace stabilization*.

Entrainment is the general phenomenon of moving the body to the pace of regular cue (such as metronome or music in the auditory sense) without specifically synchronizing each motor element to a discrete beat. This has been described as the “propensity to latch on to an [even] pulse … making human music and allied arts of dance and drill a privileged form for the exercise of our entrainment capacity” ([23], p. 7).

Entrainment is a skill that infants gradually learn. Up to the age of two, infants do not tend to adjust their movement to tempo of music with which they are engaged [24, 25]. Children then steadily improve their entrainment capacity until the age of puberty [23]. In adults, although gait characteristics are known to differ in older and younger people [26, 27], and that age-related conditions such as dementia affect entrainment ability [28], researchers have not yet directly compared entrainment in healthy younger and older adults and/or with people with Parkinson's for whom dementia as well as gait deficits are a concern as the disease progresses [29]. To recap, rhythmic entrainment is the ability of the motor system to couple with the auditory system and drive movement patterns [30]. This phenomenon can be measured using a percentage error calculation between interresponse intervals (*IRI % Error*), which compares mean sequential pacing frequencies between the cue and the movement [31, 32].

Synchronization is different to entrainment because it only occurs when the timing of self-initiated movements is simultaneously aligned to a specific point with the pacing source, a particular skill requiring the adjustment of sensorimotor reaction times using predictive timing (i.e., error correction), which enables the intentionally accurate coordination of such rhythmic behaviour in temporal synchrony (rather than intermittent or relative coordination [31]). As such, the accuracy of synchronization ability can be measured using Absolute Asynchrony, a direct comparison of the difference between the pacing event and the timed movement [33]. This skill can be trained [34], to a level of expertise (for example, in musicians [35] and dancers [36]). However, sensorimotor synchronization can also occur spontaneously; for example, when a person taps their toe or moves their head or body in time with music [37, 38]. Walking in time to the underlying beat of music does not necessarily occur as a natural phenomenon but can and does occur through explicit training [39].

In contrast to either entrainment or synchronization, *pace stability* reflects how similar each movement cycle is without direct reference to a cue source. It specifically measures within-subject movement variability using the *IRI coefficient of variance* (*IRI CoV* [40]). Compared to controls, people with basal ganglia dysfunction are sometimes more variable in their movements (e.g., [41–43]) though not always [44].

These distinctions are important as although RAS has primarily been used for gait rehabilitation, it is possible that understanding how the underlying mechanisms of these phenomena work in Parkinson's (and other pathologies) may help us extend the principles of RAS therapy to other paced movements [45, 46]. For example, metronome RAS has also been used to decrease variability in rhythmic timing of arm and finger movements [47, 48]. However, Grahn and Rowe [49] have suggested the richness of the cue may provide better guidance for movement. Furthermore, de Dreu et al. [50] and Overy [51] have suggested that there may be an additional advantage of engaging in group synchrony, in which locomotor movements are performed “in place,” i.e., as a form of dancing (or “footfall stomping” according to [23], p. 7). Dancing has been shown to ameliorate some motor (and nonmotor) symptoms for people with Parkinson's [52–54]. Dancing generally encompasses some organized rhythmic relationship between sound and movement, and understanding whether the mechanism of RAS is present (i.e., measurable in terms of entrainment and synchronization), at least at a basic level would further support these findings.

However, entrainment, synchronization, and pace stability are tested experimentally using a finger tapping synchronization-continuation task (for a review, see [31, 55]). The synchronization-continuation task begins with paced sensorimotor synchronization (i.e., tapping in time to stimuli usually for 30 secs) directly followed by a similar duration of continuous finger tapping without the stimuli (i.e., unpaced, see [40] for full theoretical description). The optimal rate for spontaneous human movement occurs in cycles between 500 and 600 ms [56], a phenomenon described as the 2 Hz human resonance theory [57] observed in various movements such as walking and clapping and also associated with a preferred tempo in music [58]. Although in general sensorimotor synchronization performance is better when the tempo of the stimuli is within this specific range, research has shown that pathology affects performance related to both the perception and production of timing [59, 60].

In Parkinson's studies specifically, Jones and Jahanshahi [6] reviewed research related to perceptual and motor timing tasks and found mixed results. The performance of people with Parkinson's was compromised in 60% of the nine perceptual timing studies analysed, 50% of time estimation tasks (two of the four studies analysed), and 67% of the time production tasks (i.e., eight of the twelve finger-tapping studies). Furthermore, tempo was an influencing factor in the studies they compared, with performance only impaired in people with Parkinson's at tapping rates faster than 500 ms. In addition to behavioural studies, Grahn and Brett [3] conducted a neuroimaging study which showed that beat perception is impaired in people with Parkinson's when comparing nonmusical beat-based stimuli to nonbeat stimuli in a discrimination task. However, Grahn [61] suggested that music may provide additional dynamic properties that may ameliorate this deficit. A further study confirmed that when the beat is embedded in musical excerpts, people with Parkinson's perform the same as controls (when ON (ON and OFF are terms commonly used to describe when a person with Parkinson's is dopaminergically medicated or not) medication [62]).

Music and metronomes offer different properties as auditory cues. Metronomes are repetitive regular nonmusical sound events experienced as a continuous stream [63]. However, they are not memorable, even by trained musicians [64]. In contrast, the underlying beat of music is memorable [65, 66]. Interestingly, in an early RAS study, Thaut et al. ([20], p. 199) reported that some people with Parkinson's reported “pacing themselves by singing the music silently” suggesting the ability to endogenously generate pacing cues in the absence of external auditory cueing. Additionally, music is known to have an effect of affective states [67], and when experienced as having “groove,” it is able to induce the urge to move [38, 68]. These properties on music may affect RAS in different ways, for example, by increasing the ability to synchronize, by helping maintain entrainment, by increasing the intention or motivation to move, by reducing perceived fatigue, or potentially by improving adherence to interventions by making “permanent cueing regimens more pleasant” [69–71]. Although music and metronomes have yet to be directly compared as pacing cues in people with Parkinson's, Leow et al. [71] did compare music with high and low groove (a subjective percept related to connection between hearing the music and wanting to move [68]), and metronomes as pacing cues for walking in neurotypical adults. The findings suggested that metronomes supported synchronization accuracy better than high-groove music, with the most asynchrony associated with low-groove music. As music has been found to be distracting in Parkinson's studies due to additional cognitive demand effects [1, 72], it would be useful to compare the effect of cue types on measures of RAS directly in people with and without Parkinson's disease.

McPherson and colleagues [73] have suggested more research is needed to understand which components (which sound cues, which types of movements, and at which tempi) produce the therapeutic effects in terms of motor rehabilitation. Therefore, the first aim of this study was to directly compare metronomes and ecologically valid music in terms of entrainment capacity (*IRI % Error*), synchronization accuracy (Absolute Asynchrony), and pacing stability (*IRI CoV*). The second aim was to compare the different types of movements in order to explore the potential for RAS beyond gait training and/or the experimental paradigm of finger tapping. To undertake this research, we devised a study in which we could compare the effect of cue types (music and metronomes in slow, medium, and fast tempi) on entrainment, synchronization, and pacing abilities in finger tapping, toe tapping, and stepping on the spot in older and younger healthy adults and people with Parkinson's. Our hypothesis were as follows:

  H_1_: music will support entrainment better than metronomes for people with Parkinson's as measured using IRI % Error across the synchronization-continuation task, particularly in the medium tempo
  H_2_: Absolute Asynchrony will be affected by tempi, and people with Parkinson's will perform significantly worse than control groups in the slow tempo metronome condition due to the deficits in their predictive timing abilities
  H_3_: people with Parkinson's will perform better when stepping on the spot in comparison with finger and toe tapping (i.e., not significantly different from controls, less IRI % Error, Absolute Asynchrony, and IRI CoV)

## 2. Methods

This study investigated the effect of cue type (music and metronome) and movement modality (finger tapping, toe tapping, and stepping on the spot) on entrainment capacity, synchronization ability, and pacing stability using a synchronization-continuation task. The synchronization-continuation task was extended with a “re-synchronization” section to provide a second set of synchronization data, thereby reducing the demand on participants with Parkinson's and also to provide an enjoyable “game-like” task with positive feedback for participants with Parkinson's in terms of their ability to engage with the different types of movements and auditory cues.

The between-subject factor was a group including people with Parkinson's (Parkinson's) and two healthy adult control groups (younger and older) with age as a potential factor based on equivocal findings in the literature. The older participants were age matched to the Parkinson's group (Section 2.1). The choice of ecologically valid music as cue types was included to differentiate from the auditory cues typically used in RAS therapy (i.e., metronomes and “rhythmically enhanced music”) so as to investigate the general use of music for people with Parkinson's as this may be more accessible in general and helpful to practitioners. The three movement modalities were chosen to enable comparison between strike-based type data from finger-tapping studies during which there is no forward motion in that type of nonspatial “tapping.” In order to find common ground between finger tapping and RAS gait studies, “stepping in the spot” represented the type of “in place” locomotion or footfall stomping previously suggested [23, 73]. The “stepping in the spot” action requires whole body movement similar to drill and incorporates that aspect of “dancing,” though with reduced degrees of freedom in movement. Tempo was an independent variable nested within stimuli (range 779–417 ms). The range of tempi was chosen to reflect the typical range of music that people move to [58, 74] but was partially constrained by choice of using only instrumental naturalistic music with a strong beat perceived in agreement through pilot testing (Section 2.2.2). This study was approved by the Health, Sciences, Engineering and Technology ECDA (Ethics Committee with Delegated Authority; Protocol Reference aLMS/SF/UH/02547) at the University of Hertfordshire. All participants provided written informed consent prior to the beginning of the study in accordance with the recommendations of the Helsinki Declaration.

### 2.1. Participants

In total, 92 participants between 18 and 80 years completed the study. The sample was split into three groups: Parkinson's: *n* = 30, 20 females, mean age = 62.23 (SD = 10.48), range 34–77 years, and the two healthy adult control groups: younger: *n* = 36, 29 females, mean age = 20.75, SD = 3.18, range 18–32 years and older (age matched to the Parkinson's group), *n* = 26, 12 females, mean age = 64.35, SD = 13.02, range 32–78 years. Participants were recruited through Parkinson's UK research network as well as through connections with the institution's Parkinson's Advisory Group and Dance for Parkinson's class. The younger group was recruited through the institution and received course credits for participation. The exclusion criteria included cognitive impairment assessed using the Mini Mental State Examination (<24 score, [75]). Participants were also asked whether they had any hearing difficulties. No participants were excluded on any of these bases.

Parkinson's group were tested during the “ON” state of their stabilized medication, and all Parkinson's participants confirmed they were diagnosed by a neurologist. The Unified Parkinson's Disease Rating Scale (UPDRS [76]) was used to evaluate their current status. The group UPDRS mean was 25.57 (SD = 10.15, range = 1–50 (max *=* 176)). Scores for the three factors that make up to overall scores were as follows: mentation, behaviour, and mood (mean = 3.5, SD = 1.68, range = 1–8 (max = 16)); activities of daily living (mean = 10.43, SD = 4.68, range = 0–21 (max = 52)); and motor examination (mean = 11.63, SD = 5.64, range = 0–25 (max = 108)). The range for this sample for the Schwab and England Activities of Daily Living Scale [77] was 50–100% and mean = 82.33% (SD = 11.94). The Hoehn and Yahr Scale [78] mean was 1.78 (SD = 0.83), ranging from 0 to 4 (max = 5). Time since diagnosis ranged from 5 months to 21 years, averaging just over 5.6 years (SD = 59.19 months). Participants were asked to report their current medication regimens. Though these data were not used in analyses, a summary of these can be found in Supplementary [Supplementary-material supplementary-material-1].

### 2.2. Equipment, Stimuli, Procedure, and Measures

#### 2.2.1. Equipment

A stomp box (used by musicians to provide bass drum sounds, generally in acoustic music) (Acoustim8, Series 100 Foot Drum, UK) was used to collect finger- and toe-tapping data in order to provide an ergonomically appropriate way of collecting tapping data and enable quick and easy transition between finger (table) and toe (floor) tapping, thereby reducing experimental demand for people with Parkinson's.

**Figure 1:**
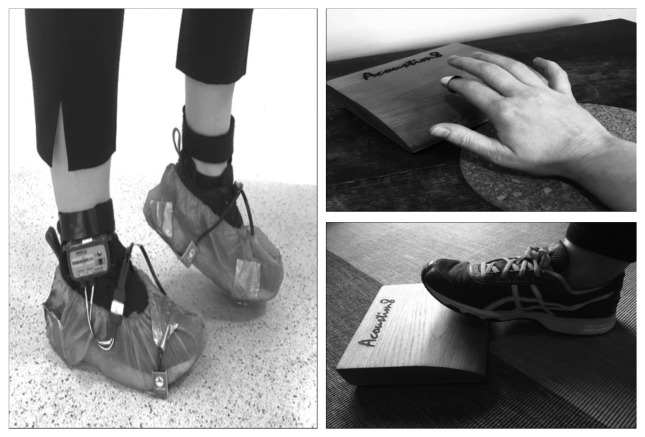
Stepping on the spot and finger- and toe-tapping equipment and actions.

BioPac heel and toe strike transducers (Model RX111) attached to BioNomadix ankle sensors (Model BN-TX STRK2-T) gathered press and release data for stepping “on the spot” (Figure 1). The experiment was ran on Superlab software (Version 5, Cedrus Corporation, San Pedro, CA) connected to an MP150 (Biopac Systems Inc., CA) unit running an STP100C Solid State Relay Drive, a UIM100C (for the StompBox), and two BioNomadix STRK2-R units (for stepping). Two mixing desks were used to split and connect the stimuli (Peavey PV6 and Behringer Xenyx502). Participants self-adjusted volume levels on headphones (Studiospares Model 448740).

#### 2.2.2. Stimuli

For the auditory cues, two types of stimuli, music and metronomes, were compared.

As dual task processing can be difficult for people with Parkinson's [1, 72], the musical stimuli chosen were instrumental excerpts (i.e., did not include any words, spoken or sung) of naturalistic music (Table 1). The metrical structure of all music was in common time (i.e., four beats in a musical bar). The aim of the music selection was to include songs that would be both familiar and unfamiliar across the participant ages (the effects of familiarity and likeability, alongside the dynamic acoustic features of the musical stimuli, are presented in a separate paper, Rose et al. [79], under review) but which also had a strong beat and included 30 second instrumental sections. To this end, 28 music excerpts were pilot tested for “ease of entrainment” prior to data collection for this study. On the basis that >60% of participants (*N* = 50) agreed that the song excerpts were “easy to tap along with,” nine songs were chosen for this study. The stimuli (including metronome beep tracks which were matched to each of the musical excerpts) were created in Logic Pro (Apple Inc., CA). In common with other timing studies, for all stimuli, an eight-beat “count in” section was provided (with an accented beep on the first and fifth beats) to reduce data loss caused by initial listening and movement adjustment by participants [31]. Stimuli were divided into slow, medium, and fast (Table 1) and analysed separately as various studies of timing in general and in Parkinson's have suggested tempo may be a mediating factor in motor abilities [6].

#### 2.2.3. Procedure

The synchronization-continuation task consisted of three consecutive sections: Sync A, Continuation Task, and Sync B (i.e., resynchronization). Participants were explicitly asked to synchronize their movements to the beat of the stimuli (i.e., either finger or toe tapping or stepping on the spot) and to try to continue that same movement when the auditory stimuli stopped, and then to resynchronize (if necessary) when the stimuli restarted. Each participant completed 18 trials (9 music and 9 metronomes), three in each movement modality. Two practice examples (first a metronome at 500 ms and then a musical example randomly assigned as either slow, medium, or fast tempo) were provided in each movement modality to ensure participants understood the task and were physically comfortable. The presentation of stimuli and movement modality were counterbalanced within-subject for each participant and between-subjects. Analysis confirmed there were no significant order effects between groups (*p* > 0.8).

#### 2.2.4. Measures

Participants were tested for beat perception ability (beat alignment test; BAT) and musical sophistication using the Goldsmiths Musical Sophistication Index (Gold MSI, [80]), both of which are freely available as research tools. The Gold MSI is a self-report scale which was developed to investigate “musical sophistication” in the general population. This term was chosen to reflect the many ways in which people can and do engage in musical activities without necessarily becoming professional musicians and therefore enable a less hierarchical, more fine-grained approach to studying the psychological effects of music and musical behaviours. The measure has been validated on 147,636 people (see Cronbach's alpha statistics in Table 2 in Section 3). The Gold BAT is presented as an online listening task during which participants state whether the click track (i.e., isochronous tone sequence) overlayed onto naturalistic instrumental music is “on” the beat (i.e., “in time with the underlying beat of the music”) or “off” the beat (i.e., asynchronous to the beat of the music). This measure has been used previously in Parkinson's research [61] and see Table 2 (Section 3) for previously unpublished score norms obtained in personal communication with Professor Müllensiefen, 2019 [81]. In this study, the general factor and the subscales of musical training and active engagement with music of the Gold MSI and the BAT scores were compared between groups in order to establish any group differences that might warrant including these factors as covariants.

### 2.3. Motor Timing Parameters

The interonset interval (IOI) refers to an audible pacing event (metronome beeps or musical beat). The interresponse interval (IRI) refers to the time interval between the onsets of two successive strikes produced by a participant. The mean IRI is commonly used to reflect the participants' capacity to accurately produce a timed motor interval [31]. The mean IOI of the stimuli classified by tempo and the mean IRI for each group are provided in Table 3.

In this study, data from the central ten bars in each of the three twelve-bar sections (Sync A, Continuation Task, and Sync B) were used in analyses. The concept of entrainment is operationalized in terms of how the auditory stimuli has been internalized by the participant, evidenced by being able to maintain their motor timing across the three consecutive sections, characterized by calculating the IRI % Error, a dependent variable demonstrating the percentage of absolute difference between each IRI and the reference IOI of a given trial (IRI % Error = (mean IRI − mean IOI)/mean IOI *∗* 100). Asynchrony is the measure of the ability to produce a rhythm that synchronizes with an expected rhythm in terms of accuracy. A second dependent variable, Absolute Asynchrony, was calculated for each strike as the time interval (in ms) between the start of the nearest sound event and the closest detected point of contact between the effectors and the tapping surface: Strike_Start_ − Pulse_Start_ (see [31, 32] for similar calculations). The third dependent variable the IRI coefficient of variation (*IRICoV*) measured within-subject performance variability (i.e., pacing stability) was calculated as IRI standard deviation/IRI mean *∗* 100) (full documentation of the data extraction can be found in Rose et al. (under review)) [79].

### 2.4. Data Preparation

During preprocessing, trials were removed from analyses if less than 18 and more than 44 strikes were recorded in all movement modalities (i.e., 10% above or below the required number of strikes). These anomalies were due to either participant error and/or equipment failure.  Table 4 shows the missing databy group, tempi, cue type, and movement modality.

Finally, a 40% criterion (deviation from interonset interval in the stimuli) was calculated to remove outliers from the *IRI* mean based on [31]. Similarly, for the Absolute Asynchrony, a 25% criterion was calculated to remove outlying data points based on [82]. This final process amounted to the loss of 7.65% data points, 7.18% for metronome stimuli, and 8.19% for music stimuli.

### 2.5. Statistical Analyses

Multifactorial repeated measures ANOVA were conducted by group (Parkinson's, older, and younger controls), across the three sections of the experimental paradigm for entrainment (measured using *IRI % Error*) and pace stability (measured using *IRI CoV*), and for Absolute Asynchrony using the data from Sync A and Sync B sections only in terms of comparing the strikes against the reference points in the auditory stimuli. Factors included cue type (metronomes and music) and movement modality (finger tapping, toe tapping, and stepping on the spot). Where significant main effects and interactions were observed, post hoc pairwise comparisons (Tukey HSD) further explore these data where the findings are considered meaningful in application for practitioners, although it is indicated when findings do not withstand Bonferroni adjustment for multiple comparisons (alpha *p* < 0.001). Where assumptions for sphericity were not met with these data, the Greenhouse–Geisser adjusted statistic is reported. The sample size required for the critical statistical test of each research hypothesis was calculated using *G* *∗* Power. Required sample size was computed for paired-samples *t* tests. In the estimation of effect size, the results of Dalla Bella et al. [12] were used as group parameters. The power analysis indicated that 18 participants minimum would be required per group (*dz* = 0.50; *α* = 0.05; 1 − *β* = 0.80). Furthermore, our sample size is similar to that used in other Parkinson's studies (e.g., [12] (*N* = 21), [44] (*N* = 15), [83] (*N* = 18), [20] (*N* = 15), and [84] (*N* = 22)). Effect sizes are reported as partial eta squared (interpreted as small = 0.01, medium = 0.06, and large = 0.14 according to [85, 86]). Analyses were conducted with SPSS software (v23, IBM Inc.).

## 3. Results

### 3.1. Goldsmiths Beat Alignment Test (BAT) and Musical Sophistication Index (MSI)

No significant between-group differences were found for the Gold MSI general measure (*p* > 0.05), or for the subscales of musical training (*p* > 0.1), and active engagement with music (*p* > 0.1), or for the Gold BAT (*p* > 0.5) (Table 2). As the range of scores was similar to the published population norms, these data were not included in further analyses for this study.

### 3.2. Entrainment, Synchronization, and Pacing Stability

Table 5 presents the results for the multifactorial repeated measures ANOVA for the dependent variables (IRI % Error, *IRICoV*, and Absolute Asynchrony).

#### 3.2.1. Entrainment: IRI % Error

**Figure 2:**
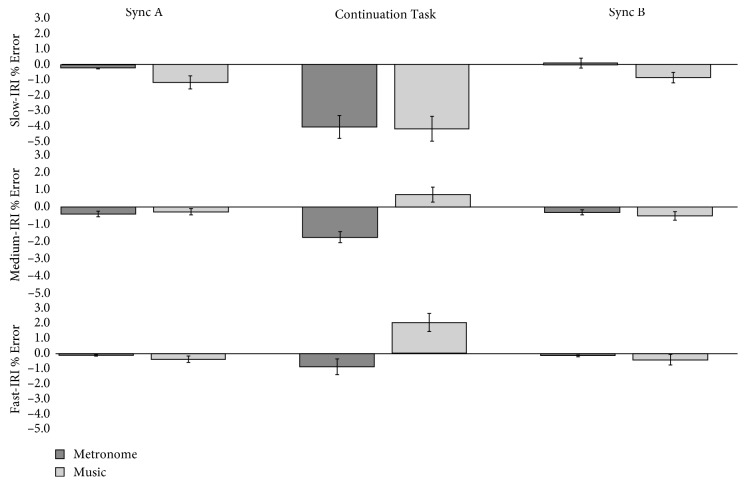
The main effect of cue type on entrainment ability (IRI % Error) collapsed across all groups in each tempo. *Y*-axis shows IRI % Error. *X*-axis shows the temporal nature of the experimental paradigm.

*(1) Cue Type*. In slow tempo, no main effect or group interactions were revealed. In medium tempo, a significant main effect showed that overall participants performed with less error (i.e., closest to 0, *p*=0.002) with music (mean IRI % Error = −0.003, SE = 0.233) compared to metronome (mean IRI % Error = −0.799, SE = 0.152). The mean difference between cue types was ±0.796, ms, *p*=0.002, and CI ± 0.315–1.276. The effect size of this result was large, and as Figure 2 shows the direction of error differed for metronome (negative) and music (positive). No interaction between groups was revealed in this tempo. In fast tempo, a significant main effect showed that overall participants performed with less error with metronome than with music. Pairwise comparisons showed the mean difference between cue types in the fast tempo was ±0.793 ms, *p*=0.001, and CI ± 0.361–1.225. The metronome mean was negative at −0.352 ms and SE = 0.198, whereas the music was positive, 0.441 ms and SE = 0.347. Figure 2 illustrates how the effect of auditory cueing is most observable during the continuation task section of the experimental paradigm.

Repeated measures ANOVA results revealed the following:

  For slow tempo, no effect main effect (*p* > 0.1) or group interaction (*p* > 0.3)
  For medium tempo, a main effect *F*(1, 62) = 10.966, *p*=0.002, and *η*_*ρ*_^2^ = 0.150, and no group interaction (*p* > 0.2)
  For fast tempo, a main effect *F*(1, 59) = 13.512, *p*=0.001, and *η*_*ρ*_^2^ = 0.186 and a group interaction *F*(2, 59) = 3.391, *p*=0.040, and *η*_*ρ*_^2^ = 0.103
  Confidence intervals = standard error

**Figure 3:**
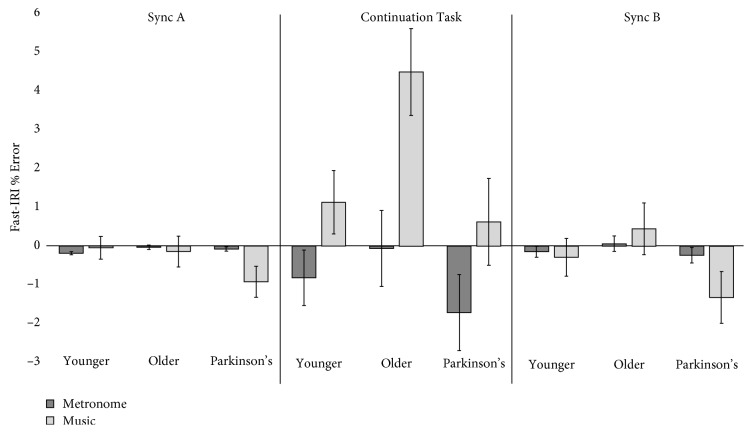
The significant main effect of cue type on entrainment ability (IRI % Error) and the significant interaction between groups in the fast tempo. *Y*-axis shows IRI % Error. *X*-axis shows the temporal nature of the experimental paradigm.

Analysis of the significant interaction between groups in the fast tempo revealed a significant difference between the older and Parkinson's groups (±1.395 ms, *p*=0.050, and CI ± 0.0–2.790) as illustrated in Figure 3. The Parkinson's and younger groups did not differ significantly (*p* > 0.3), and neither did the older and younger groups (*p* > 0.1). Although the effect size for this interaction was large, it should be noted that the result does not withstand Bonferroni adjustment for alpha *p*.

Repeated measures ANOVA results revealed the following:

  A main effect of cue type *F*(1, 59) = 13.512, *p*=0.001, and *η*_*ρ*_^2^ = 0.186
  An interaction between groups, *F*(2, 59) = 3.391, *p*=0.040, and *η*_*ρ*_^2^ = 0.103
  Confidence intervals = standard error

**Figure 4:**
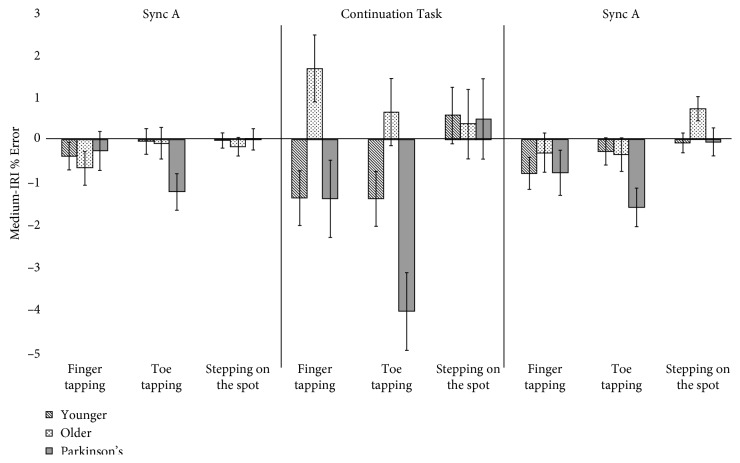
The significant main effect of movement modality on entrainment ability (IRI % Error) and the significant interaction between groups in the medium tempo. *Y*-axis shows IRI % Error. *X*-axis shows the temporal nature of the experimental paradigm.

*(2) Movement Modality*. No main effects or interactions were found for the slow tempo (*p* > 0.1, *p* > 0.3). In the medium tempo, a significant main effect showed that modality effected entrainment. Overall, participants performed best when stepping in the spot (mean = 0.200, SE = 0.200), followed by finger tapping (mean = −0.476, SE = 0.242), and least well when toe tapping (mean = −0.926, SE = 0.256). Significant differences were revealed between toe tapping and stepping on the spot (±1.126, *p* < 0.001, and CI ± 0.60–1.652) and between finger tapping and stepping on the spot (±0.676, *p*=0.043, and CI ± 0.022–1.33) but not between the two types of tapping (*p* > 0.1). A significant interaction between groups was driven by a difference between the Parkinson's and older participants (mean diff ± 1.179, *p*=0.016, and CI ± 0.189–2.168).  Figure 4 illustrates how people with Parkinson's made the most errors when toe tapping and the least for stepping on the spot and that the older group was the most consistent (i.e., errors closest to 0) for all three movement modalities in the medium tempo. No significant differences were revealed between Parkinson's and the Younger group performances (*p* > 0.3), or between controls groups (*p* > 0.1). However, the interaction statistic does not withstand Bonferroni adjustment for alpha *p*. In the fast tempo, post hoc analysis of the main effect revealed a mean difference between finger tapping and stepping on the spot (±1.548, *p*=0.009, and CI ± 0.208–2.005), and toe tapping and stepping on the spot (±.649, *p*=0.005, and CI ± 0.207–1.091), but not between finger and toe tapping (*p* > 0.1). Overall in the fast tempo, participants performed with negative error when finger tapping (mean = −0.771, SE = 0.590), closest to 0 when toe tapping (mean = 0.128, SE = 0.241) and with positive error when stepping on the spot (mean = 0.777, SE = 0.163).

Repeated measures ANOVA results revealed the following:

  A main effect of modality *F*(2, 124) = 7.035, *p*=0.001, and *η*_*ρ*_^2^ = 0.102
  An interaction with group, *F*(4, 124) = 2.629, *p*=0.038, and *η*_*ρ*_^2^ *=* 0.078
  Confidence intervals = standard error

#### 3.2.2. Synchronization: Absolute Asynchrony

**Figure 5:**
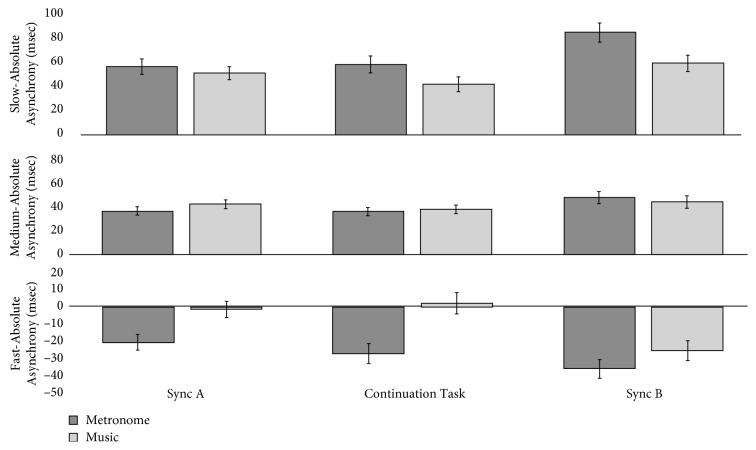
The effect of cue type on Absolute Asynchrony in all tempi by group. Y-axis shows the mean of Sync A and Sync B Absolute Asynchrony in ms. X-axis shows group by cue type.

*(1) Cue Type*. A main effect of cue type was revealed in the slow tempo. Significantly more Absolute Asynchrony was evident in the metronome condition (mean = 66.265 ms, SE = 4.130) compared to the music condition (mean = 50.433 ms, SE = 3.519). Post hoc tests show a mean difference between cue type was ±15.832 ms, *p* < 0.001, and CI ± 9.635–22.029. A significant interaction between groups was also revealed, although the *p* value did not withstand Bonferroni adjustment for multiple comparisons. Pairwise comparisons confirmed this; Absolute Asynchrony did not differ between Parkinson's and older groups (*p*=0.051), Parkinson's and the younger group (*p* > 0.1), nor between control groups (*p* > 0.8) in the slow tempo. In the medium tempo, no main effect of cue type (*p* > 0.6) or interaction between groups (*p* > 0.5) was revealed. A main effect of cue type was revealed in the fast tempo. Significantly more Absolute Asynchrony was evident in the metronome condition (mean = −27.488 ms, SE = 3.025) compared to the music condition (mean = −7.940 ms, SE = 3.187). Post hoc tests show a mean difference between cue type was ±19.548 ms, *p* < 0.001, and CI ± 13.580–25.516 ms. No interaction between groups was found in the fast tempo (*p*=0.068).  Figure 5 illustrates the general effect of cue type on synchronization ability in all tempo.

Repeated measures ANOVA results revealed the following:

  Slow tempo: a main effect of cue type *F*(1, 43) *=* 26.544, *p* < 0.001, and *η*_*ρ*_^2^ *=* 0.382and an interaction with group, *F*(2, 43) *=* 3.692, *p*=0.033, and *η*_*ρ*_^2^ = 0.147
  Medium tempo: no significant main effect (*p* > 0.6) or interaction between groups (*p* > 0.5).
  Fast tempo: a main effect of cue type *F*(1, 47) *=* 43.417, *p* < 0.001, and *η*_*ρ*_^2^ = 0.480, and no interaction, *p*=0.068
  Confidence intervals = standard error

*(2) Movement Modality*. In the slow tempo, a significant main effect of modality on Absolute Asynchrony was revealed. The most errors occurred when toe tapping (mean = 72.419 ms, SE = 4.436), followed by finger tapping (mean = 60.370 ms, SE = 4.172), and the least when stepping on the spot (mean = 42.258 ms, SE = 4.489). Pairwise comparisons showed that the difference between finger tapping and toe tapping was significant (±12.049 ms, *p*=0.005, and CI ± 3.855–20.243), the difference between finger tapping and stepping on the spot was significant (±18.112 ms, *p* < 0.001, and CI ± 8.471–27.754), and the difference between toe tapping and stepping on the spot was also significant (±30.161 ms, *p* < 0.001, and CI ± 20.901–39.421). There was no interaction between groups (*p* > 0.3).

**Figure 6:**
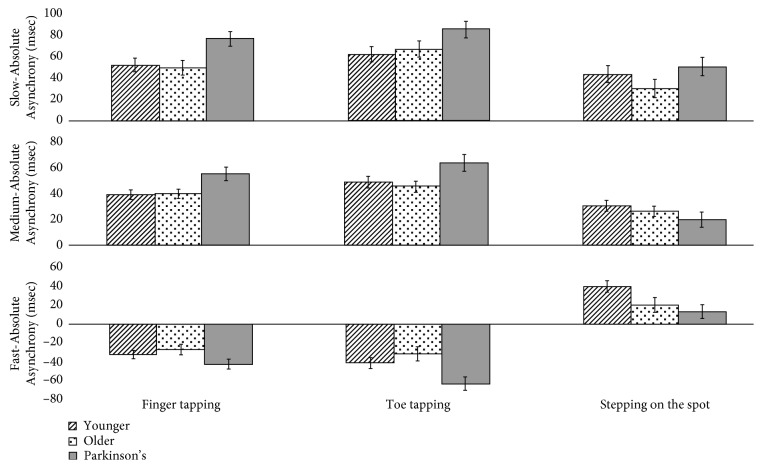
The effect of movement modality on Absolute Asynchrony in all tempi by group. *Y*-axis shows Absolute Asynchrony in ms. *X*-axis shows group by movement modality.

In the medium tempo, a significant main effect of modality was also revealed. Pairwise comparisons showed the overall mean difference between finger tapping and toe tapping was ±7.841 ms, *p*=0.01, and CI ± 1.992–13.690; between finger tapping and stepping on the spot, ±19.077, *p* < 0.001, and CI ± 11.436–26.718; and between toe tapping and stepping on the spot, ±26.918, *p* < 0.001, and CI ± 19.405–34.431. Although an interaction between groups was revealed, it did not withstand Bonferroni correction. Post hoc pairwise comparisons confirmed groups did not significantly differ (Parkinson's and older: *p*=0.065; Parkinson's and younger: *p* > 0.1; and controls *p* > 0.5). For people with Parkinson's, stepping on the spot produced the best results in terms of the least asynchrony (20 ms), followed by finger tapping (39 ms), whereas toe tapping produced the most asynchrony (48 ms) (Figure 6). For the fast tempo, a main effect of modality was revealed showing that overall, participant performed the most asynchrony when toe tapping (mean = −44.931 ms, SE = 3.926), followed by finger tapping (mean = −33.398 ms, SE = 2.957), and the least when stepping on the spot (mean = 25.186 ms, SE = 4.129). Pairwise comparisons showed that the difference between finger tapping and toe tapping was significant (±11.533 ms, *p*=0.003, and CI ± 4.167–18.900), the difference between finger tapping and stepping on the spot was significant (±58.584 ms, *p* < 0.001, and CI ± 50.342–66.826), and the difference between toe tapping and stepping on the spot was also significant (±70.118 ms, *p* < 0.001, and CI ± 59.795–80.441). The between-group interaction analyses showed a significant difference between the Parkinson's and younger group (±19.574 ms, *p*=0.009, and CI ± 4.309–34.838) and also between the Parkinson's and older group (±18.112 ms, *p*=0.038, and CI ± 0.838–35.386). The control groups did not differ significantly (*p* > 0.9). However, this result did not withstand Bonferroni correction.

Repeated measures ANOVA results revealed the following:

  Slow tempo: a main effect of modality *F*(2, 86) *=* 22.879, *p* < 0.001, and *η*_*ρ*_^2^ *=* 0.347, and no interaction between groups (*p* > 0.3)
  Medium tempo: a main effect of modality *F*(2, 66) *=* 31.938, *p* < 0.001, and *η*_*ρ*_^2^ *=* 0.492, and an interaction between groups *F*(4, 66) *=* 3.089, *p* > 0.022, and *η*_*ρ*_^2^ *=* 0.158
  Fast tempo: a main effect of modality *F*(2, 94) *=* 150.058, *p* < 0.001, and *η*_*ρ*_^2^ *=* 0.761, and an interaction between groups *F*(4, 94) *=* 2.818, *p*=0.038, and *η*_*ρ*_^2^ *=* 0.107
  Confidence intervals = standard error

#### 3.2.3. Pacing Stability: IRICoV

*(1) Cue Type*. In the slow tempo, analyses of *IRICoV* across all three sections of the experimental paradigm revealed no main effect of cue type (*p* > 0.7) and no interaction between groups (*p* > 0.5). A main effect of cue type was revealed in the medium tempo. The mean difference between metronome and music was ±2.197 ms, *p*=0.011, and CI ± 0.511–3.883 with more variance observed for music than for metronome (Table 6). No interaction between groups was revealed in this tempo (*p* > 0.9). In the fast tempo, a main effect of cue type was revealed with a mean difference of ±1.439 ms, *p*=0.011, and CI ± 0.344–2.533, with more variance observed in the music condition compared to the metronome (Table 6). There were no interactions between groups in fast tempo (*p* > 0.1). The effect of cue type on *IRICoV* in the medium and fast tempi did not withstand Bonferroni correction for multiple comparisons.

Table 6 shows data relating to IRI CoV for cue type and movement modality.

*(2) Movement Modality*. In all tempi, significant main effects were revealed in *IRICoV*, and no interactions between groups (Table 6). In slow tempo, the mean difference between finger tapping and toe tapping was significant ±4.099 ms, *p*=0.015, and CI ± 0.826–7.371, and between finger tapping and stepping on the spot ±12.772 ms, *p* < 0.001, and CI ± 8.748–16.797, and also between toe tapping and stepping on the spot ±16.871 ms, *p* < 0.001, and CI ± 12.096–26.646. In the medium tempo, there was a significant mean difference between finger tapping and stepping on the spot was ±12.711 ms, *p* < 0.001, and CI ± 9.711–15.710, and between toe tapping and stepping on the spot ±14.449 ms, *p* < 0.001, and CI ± 11.100–17.799. The difference between finger tapping and toe tapping was not significant (*p*=0.08). In the fast tempo, the mean difference between finger tapping and stepping on the spot was ±12.386 ms, *p* < 0.001, and CI ± 10.510–14.260, and also between toe tapping and stepping on the spot ±13.818 ms, *p* < 0.001, and CI ± 11.587–16.049. The difference between finger tapping and toe tapping was not significant (*p*=0.09).

## 4. Discussion

This study investigated how different sound cues and different types of movements affected rhythmical motor behaviours at different tempi in people with and without Parkinson's. Overall, the findings suggest that (a) music helps people with Parkinson's maintain entrainment better than metronomes and (b) that stepping on the spot enables people with Parkinson's to entrain better than either finger or toe tapping. We also note that our results suggested that age did not effect entrainment, synchronization, or pacing stability. Specifically, in relation to our first hypothesis, music did support entrainment better than metronomes, as measured using IRI % Error in the medium but also in the fast tempo. The effect of entrainment was especially noticeable during the continuation task as illustrated in Figure 2. This will be discussed in Section 4.1, in relation to priming and the potential for therapeutic use of imagined music. It was also notable that people with Parkinson's did not differ from controls, even in the slow tempo, and that they were able to resynchronize (i.e., latch on to the beat again in Sync B) as successfully as controls. With regard to our second hypothesis, tempi did affect the results for Absolute Asynchrony, and all groups, not just the people with Parkinson's performed worse (i.e., with significantly more asynchrony) in the slow tempo condition. Figures 5 and 6 illustrate the change in direction of asynchrony error in the fast tempo. It is notable that negative error is only performed in the fast tempo when tapping, and not stepping on the spot and this will be discussed further in Section 4.2 in relation to ideas concerning emergent timing. This point is related to our final hypothesis, for which we confirmed that stepping on the spot enabled better timed motor behaviour for all measures compared to tapping. This has important implications for research in understanding how embodied entrainment may differ from effector entrainment and may also be connected to emergent timing. Therapeutically, these findings suggest that RAS training could be used for other types of movements that may be used to improve functional mobility for people with Parkinson's. These results are discussed in the following sections.

### 4.1. Music Effect

Overall, the type of cue did not affect pacing stability, yet music reduced asynchrony in the slow and fast tempo (there was no difference in the medium tempo). These findings suggests that, in this sample, the music did not create a demand effect for people with Parkinson's as was found in Brown and Marsden [1] and Brown et al., [72], although this was a different type of task. Furthermore, although it did not matter in terms of synchronization ability which cue type was heard in the medium tempo, in the slow and fast tempi conditions, the music more than the metronomes helped people with Parkinson's to synchronize as well as controls. This provides useful baseline information for practitioners in terms of using music to help engage people with Parkinson's in movements programmes. For example, fatigue is a common symptom of Parkinson's [87], but music has been shown to promote ergogenic effect (i.e., reduce the perception of fatigue to enable continued exercise) [69, 70]. However, practitioners should take care to individualize musically enhanced rehabilitation programmes as, although most participants with Parkinson's in this study anecdotally reported enjoying the music more than the metronomes, some reported feeling that the music “pushed them out of the way.” Although no significant differences were found in the Gold Beat Alignment Test, a more extensive measure of rhythmic perception and production abilities, such as the BAASTA [88], may have revealed more fine-grained differences. As suggested in Dalla Bella et al. [12], individual differences in rhythmic perception and production abilities may be a fundamental aspect with regard to the usefulness of music in terms of external rhythmic auditory guidance.

The findings relating to the phenomenon of entrainment in this study showed that music had a much larger effect than metronomes during the continuation task for all participants in terms of maintaining entrainment in the absence of heard cues (i.e., during the continuation task). In this study, similar to the comments reported in Thaut et al. [20], several participants explained that they maintained entrainment by singing the music inside their minds. For example, one participant with Parkinson's explained “The beat was like a shadow inside my head, but I could keep singing along with the music.” and another reflected, “The problem with metronome was that once you lost it, there was no way to find your way back.” These, and other similar comments regarding strategies involving subvocalization, suggest that understanding what occurs between paced and unpaced motor timing may have useful application in Parkinson's rehabilitation. It could be that the repetition of rhythmic musical phrases (including melodies, with or without lyrics) induces a priming effect than can be further enhanced with training. Not only is the underlying beat of music memorable [64–66], studies investigating the phenomenon and prevalence of “sticky tunes” or “earworms” (91.7% of people experience a weekly earworm [89, 90]) suggest our musical imaginations can be triggered by two musical features common in RAS therapy; repetition and musical simplicity. Similarly, the familiarity and likeability of the music is important and will be considered in a follow-up paper. Schaefer and colleagues [91] have suggested that heard and imagined music can modulate movement in subtly different ways. In their fMRI study (with neurotypical participants using a wrist inflection movement task), Schaefer and colleagues showed that when listening to heard music, more activation was observed in the cerebellum, whereas when listening to imagined music more activation was observed in the presupplementary motor area. The phenomenon of endogenous timing strategies (as opposed to spontaneous motor tempo [42]) has been described as a form of “covert, internal synchronization” ([31], p. 969), whereby people generate temporal expectations from the rhythm of what they have been listening to. Clayton [92] described the phenomena as intraindividual entrainment. This suggests that the music itself is a form of priming, and that in turn RAS therapy co-opts this as a form of training, explaining to some extant to reports of “carry over effects,” i.e., the continued effects of RAS training on gait for some weeks, or even months posttraining. In order to extend RAS training beyond the reliance on the continuous presentation of stimuli [44, 93], further research is required to develop strategies to harness the musical imagination in the form of RAS therapy. Only one study has shown that imagined music can help walking for people with Parkinson's [94], but the present study provides support for the supposition of Schaefer and colleagues [91, 95] who also suggested the impact of the cue may depend on the type of movement.

### 4.2. Movement Effect

The findings of this study also demonstrated that the stepping on the spot task was better than finger tapping, and especially toe tapping, in terms of entrainment, synchronization, and pace stability for all participants. Importantly, this type of stationary “walking” enabled people with Parkinson's to perform at the same level as control groups, showing that to some extant, the principles of RAS training extend to other types of movements. In a similar way that people with Parkinson's reported music mostly enabled subvocalizing, the stepping on the spot task was described as an easy and natural movement in comparison to tapping, especially toe tapping. As one participant described, “I just let my body do the movement. It felt natural, and when I knew the song, it was easy to keep it going inside my head.”

This is pertinent in relation to Parkinson's because although entrainment, though also considered as a neural oscillatory process [96], is managed behaviourallyin part by the “afferent feedback of the movement”. This in turn is thought to be involved in the anticipatory processes necessary for sensorimotor synchronization ([95], p. 3). The accumulation from the different sensory channels is embedded in the sensory accumulator model [33, 97]. Leman and Maes [98] described the way in which the sensorimotor networks in the human body mediate the affective experience of music as embodied music cognition. The findings herein suggest that whole body continuous movement (i.e., stepping on the spot) helps people with Parkinson's to entrain, synchronize, and pace better than more discrete effector movements such as tapping. Therefore, we suggest the term *embodied entrainment* to describe this phenomenon.

The findings relating to the differences in movement modalities are also important because of the overlap between event-based timing and discrete movements and emergent timing and continuous movement (e.g., [99, 100]). Ivry and Richardson [101] suggested a multiple timer model speculating on the characteristic functional roles of the basal ganglia and cerebellum in timing. However, when comparing the models using stimuli set at a rate of 550 ms, Spencer and Ivry [7] did not find any group differences in their Parkinson's study which the authors suggested was due to the relatively spared effector control in their Parkinson's sample. Interestingly, a recent study [102] suggests that there is a transition between these two modes of timing at 600 ms (whereby a reliance on event-based timing is observed < 600 ms). In the present study, the findings show a switch in the direction of error (from negative to positive (Figure 6)) specific to stepping on the spot in the fast tempo, but not tapping which remained negative. This suggests that the emergent timing processes involved in continuous movement may enable people with Parkinson's to engage with motor actions at a faster pace. This information may be useful in therapeutic application when considering which movements to rehabilitate at which speeds.

### 4.3. Limitations

Although extension of the synchronization-continuation paradigm provided two sets of data measuring asynchrony, and the novel use of equipment did reduce participant demand, we acknowledge that even with this large sample of people with Parkinson's, the findings reported herein will require replication in order to be considered robust. Furthermore, although we chose to use each musical excerpt only once to ensure learning effects did not occur during the experiment that does not necessarily mean that participants were more able to entrain with more familiar musical stimuli, or at stimuli closer to their own spontaneous motor tempo. However, these questions will be addressed in a separate paper. Moreover, we acknowledge that the choice of movement modalities was not directly comparable in that stepping on the spot is an interlimb coordinated movement, whereas finger and toe tapping require rather more cognitive attention. However, the requirement for participants to stay in one spot (rather than walk with forward trajectory) did require some adjustment for some participants. Future studies may consider using motion capture technology to compare the timing, amplitude, and synchrony of movements pre- and postrehabilitation programmes. Finally, we acknowledge that including participant commentary as insight for strategies for behaviour is not sufficient in terms of the requirements for data. However, we believe the inclusion of the voice of the participants with Parkinson's is a necessary and valuable contribution and in line with the guidance for patient and public involvement provided by Parkinson's UK.

### 4.4. Future Directions

The efficacy of many interventions relies on adherence to the therapeutic programme and ideally continued practice to maintain training effects afterwards [10]. Music provides an engaging auditory stimuli, which research in sports and exercise science has shown in itself has an energizing and/or motivating effect on movement [70]. For example, the experience of “groove” as inducing the pleasurable urge to move, as well as other dynamic acoustic features [103], may shed light on the mechanisms by which music supports entrainment [14, 34, 73]. Moreover, although current research focusing on the neural mechanisms of timing is essential, studies considering the potential of heard and imagined music (both primed and self-generated) and remembered music could be designed in parallel. For example, the role of musical memory, especially autobiographical memory, has also yet to be explored and utilized in people with Parkinson's as it has successfully in other associated pathologies (e.g., music and dementia [104, 105]). Salient musical memories may also be associated with movements, and in particular with dancing [51, 53, 106, 107]. As Schaefer [95] has commented, the impact of the cue may depend not only on the type of movement involved but also on the salient strength of the music (whether perceived externally or represented internally) as the phenomena of entrainment and synchronization rely on both conscious and unconscious processes.

## 5. Conclusion

This is the first study to demonstrate that people with Parkinson's can entrain as well as control when primed by music rather than metronomes beeps. We suggest that when using the body to produce timed sequences of action (herein operationalized as stepping on the spot rather than finger or toe tapping), people with Parkinson's can reach performance levels as accurately and with as much stability as those observed in healthy individuals. This is especially true when using music as the pacing cue. Music may trigger body dynamics and facilitate the emergence of embodied timing, which requires less cognitive control than predictive timing. These findings provide possibilities for direct application to therapeutic approaches for motor rehabilitation to help people with Parkinson's learn to use alternative strategies. As such, learning to entrain to an inner jukebox of tunes may help people with Parkinson's learn to manage movement better and therefore reduce the risks of falls.

## Figures and Tables

**Table 1:** Naturalistic musical stimuli.

Song code	Tempo	Beats per minute	Interbeat interval (ms)	Song	Artist	Year of release
	Slow^a^	69	870	Moments in Love	Art of Noise	1984
Song 1	Slow	77	779	Teardrop	Massive Attack	1998
Song 2	Slow	81	741	El Condor Pasa	Leo Rojas	2012
Song 3	Slow	85	706	Bitter Sweet symphony	The Verve	1997
	Medium^a^	120	500	España Cañí	Pascual Marquina Narro	1923 (recording 2010)
Song 4	Medium	112	536	Robot Rock	Daft Punk	2005
Song 5	Medium	117	513	Axel F	Harold Faltermeyer	1984
Song 6	Medium	120	500	March of Toreadors from Carmen	Georges Bizet	1875 (recording 2011)
	Fast^a^	125	480	Get Ready for This	2 unlimited	1991
Song 7	Fast	136	441	Material Girl	Madonna	1984
Song 8	Fast	139	432	Beat It	Michael Jackson	1983
Song 9	Fast	144	417	The Beautiful People	Marilyn Manson	1996

^a^Used for practice trials only.

**Table 2:** Goldsmiths Beat Alignment Test and Musical Sophistication Index by group.

	Younger			Older			Parkinson's			Gold MSI Population Norms			
	Mean	SD	Range	Mean	SD	Range	Mean	SD	Range	Mean	SD	Range	Cronbach's alpha
Beat Alignment Test Scores	10.66	1.43	8–14	11.31	2.59	6–16	11.03	2.59	7–16	11.98	2.80	Chance = 8.5/17	0.67
*Music Sophistication Index*													
General	69.78	15.58	35–99	68.96	22.57	31–114	59.70	15.55	33–95	81.58	20.62	18–126	0.93
Musical Training Subscale	19.75	8.27	7–36	21.54	12.37	7–43	16.13	9.75	7–39	26.52	11.44	7–49	0.90
Active Engagement Subscale	36.50	9.41	18–53	34.38	11.85	15–57	31.77	7.80	16–44	41.52	10.36	9–63	0.87

**Table 3:** Mean interonset interval of the stimuli and interresponse intervals by group for each tempo.

Tempo	Beats per minute	IOI	IRI younger mean^a^ (SD)	IRI older mean^a^ (SD)	IRI Parkinson's mean^a^ (SD)
Slow	81.02	741.87	740.35 (31.77)	735.86 (36.14)	735.15 (52.89)
Medium	116.25	516.71	515.56 (17.10)	516.63 (19.46)	514.10 (19.33)
Fast	139.68	429.95	430.12 (10.79)	430.21 (9.89)	428.35 (14.98)

IOI, interonset interval; IRI, interresponse interval; SD, standard deviation; ^a^milliseconds.

**Table 4:** Missing data by group, tempi, cue type and movement modality.

	Metronome			Music		
	Finger tapping	Toe tapping	Stepping on the spot	Finger tapping	Toe tapping	Stepping on the spot
Slow tempo						
Younger	0	2	1	0	2	3
Older	0	6	4	0	6	4
Parkinson's	1	2	5	1	1	3
Total *N* missing	1	10	10	1	9	10
% missing	1.09	10.87	10.87	1.09	9.78	10.87
Medium tempo						
Younger	0	2	1	0	2	2
Older	0	1	3	0	1	4
Parkinson's	0	5	7	1	4	5
Total *N* missing	0	8	11	1	7	11
% missing	0.00	8.70	11.96	1.09	7.61	11.96
Fast tempo						
Younger	1	2	3	0	1	1
Older	0	1	2	0	1	7
Parkinson's	0	4	9	1	8	5
Total *N* missing	1	7	14	1	10	13
% missing	1.09	7.61	15.22	1.09	10.87	14.13
Overall *N* missing	2	25	35	3	26	34
Overall % missing	0.72	9.06	12.68	1.09	9.42	12.32

**Table 5:** Repeated measures ANOVA results for modality and cue type by tempi.

	IRI % Error	IRICoV	Absolute Asynchrony
*Cue type*			
Slow			
Main effect	ns, *p* > 0.1	ns, *p* > 0.7	*F*(1, 43) *=* 26.544, *p* < 0.001, *η*_*ρ*_^2^ = 0.382
Group interaction	ns, *p* > 0.3	ns, *p* > 0.5	*F*(2, 43) *=* 3.692, *p*=0.033, *η*_*ρ*_^2^ = 0.147
Medium			
Main effect	*F*(1, 62) *=* 10.966, *p*=0.002, *η*_*ρ*_^2^ *=* 0.150	*F*(1, 62) *=* 6.785, *p*=0.011, *η*_*ρ*_^2^ *=* 0.099	*p* > 0.6
Group interaction	ns, *p* > 0.2	ns, *p* > 0.9	*p* > 0.5
Fast			
Main effect	*F*(1, 59) *=* 13.512, *p*=0.001, *η*_*ρ*_^2^ *=* 0.186	*F*(1, 59) *=* 6.918, *p*=0.011, *η*_*ρ*_^2^ *=* 0.105	*F*(1, 47) *=* 43.417, *p* < 0.001, *η*_*ρ*_^2^ = 0.480
Group interaction	*F*(2, 59) = 3.391, *p*=0.040, *η*_*ρ*_^2^ = 0.103	ns, *p* > 0.1	ns, *p*=0.068

*Modality*			
Slow			
Main effect	ns, *p* > 0.1	*F*(2, 128) *=* 37.299, *p* < 0.001, *η*_*ρ*_^2^ *=* 0.368	*F*(2, 86) *=* 22.879, *p* < 0.001, *η*_*ρ*_^2^ = 0.347
Group interaction	ns, *p* > 0.3	ns, *p* > 0.7	ns, *p* > 0.3
Medium			
Main effect	*F*(2, 124) *=* 7.035, *p*=0.001, *η*_*ρ*_^2^ = 0.102	*F*(2, 124) *=* 61.920, *p* < 0.001, *η*_*ρ*_^2^ *=* 0.500	*F*(2, 66) *=* 31.938, *p* < 0.001, *η*_*ρ*_^2^ *=* 0.492
Group interaction	*F*(4, 124) *=* 2.629, *p*=0.038, *η*_*ρ*_^2^ *=* 0.078	ns, *p*=0.082	*F*(4, 66) *=* 3.089, *p*=0.022, *η*_*ρ*_^2^ *=* 0.158
Fast			
Main effect	*F*(2, 118) *=* 5.312, *p*=0.018, *η*_*ρ*_^2^ *=* 0.083^a^	*F*(2, 118) *=* 121.478, *p* < 0.001, *η*_*ρ*_^2^ *=* 0.673	*F*(2, 94) *=* 150.058, *p* < 0.001, *η*_*ρ*_^2^ = 0.761
Group interaction	ns, *p* > 0.2	ns, *p* > 0.5	*F*(4, 94) *=* 2.818, *p*=0.038, *η*_*ρ*_^2^ = 0.107

^a^Values reporting a Greenhouse‐Geisser statistic.

**Table 6:** IRI coefficient of variation for slow, medium, and fast tempi by movement modality.

Tempo	Cue type	Mean (ms)	Std. error (ms)	95% confidence interval	
				Lower bound	Upper bound
Slow	Metronome	36.24	1.72	32.82	39.67
	Music	35.71	1.34	33.04	38.38
Medium	Metronome	25.27	0.89	23.50	27.04
	Music	27.47	1.11	25.25	29.69
Fast	Metronome	20.94	0.70	19.54	22.35
	Music	22.38	0.69	21.00	23.76

	Modality				

Slow	Finger tapping	38.87	1.52	35.83	41.91
	Toe tapping	42.97	2.04	38.89	47.04
	Stepping on the spot	26.10	1.77	22.56	29.63
Medium	Finger tapping	30.03	0.97	28.08	31.98
	Toe tapping	31.77	1.27	29.24	34.30
	Stepping on the spot	17.32	1.40	14.53	20.11
Fast	Finger tapping	25.31	0.85	23.61	27.02
	Toe tapping	26.75	0.94	24.86	28.63
	Stepping on the spot	12.93	0.75	11.43	14.43

## Data Availability

The data used to support the findings of this study are available from the corresponding author upon request.

## Supplementary Materials

Supplementary Materials Supplementary Table S1: an overview of medication regimens for Parkinson's as reported by participants with Parkinson's.
