# Supplementary figures and images for: Music and Metronomes Differentially Impact Motor Timing in People with and without Parkinson's Disease: Effects of Slow, Medium, and Fast Tempi on Entrainment and Synchronization Performances in Finger Tapping, Toe Tapping, and Stepping on the Spot Tasks
Source: Parkinsons Dis. 2019 Aug 18;2019:6530838. doi: 10.1155/2019/6530838 (PMC6721399; doi:10.1155/2019/6530838)

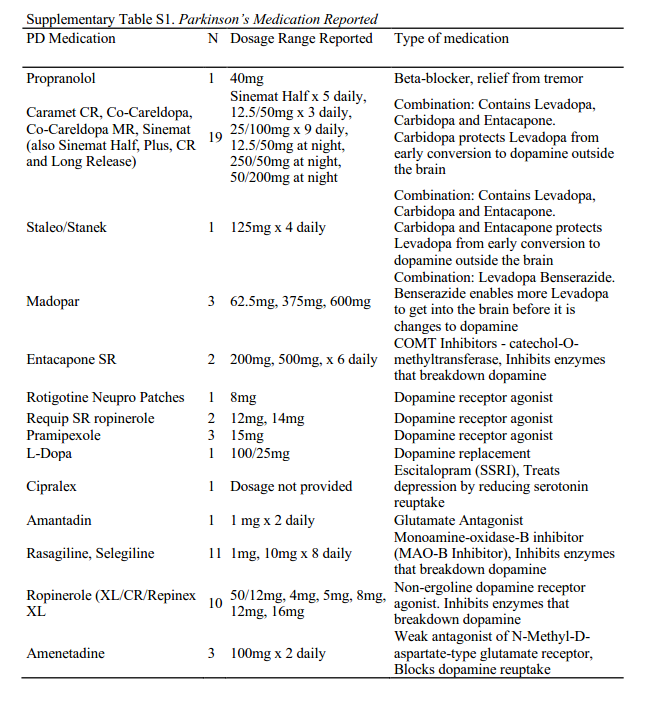

Supplement: Supplementary Materials — Supplementary Table S1: an overview of medication regimens for Parkinson's as reported by participants with Parkinson's. [file 6530838.f1.docx]
